# Effects of glargine on hyperglycemia in patients with diabetes mellitus type II undergoing off-pump coronary artery bypass graft: A randomized, controlled, double-blind clinical trial

**DOI:** 10.34172/jcvtr.2023.31596

**Published:** 2023-03-16

**Authors:** Shima Sheybani, Mahdi Kahrom, Raheleh Ganjali, Seyedeh Mahsa Kalati, Nahid Zirak, Vahideh Ghorani

**Affiliations:** ^1^Department of Anesthesiology, Faculty of Medicine, Mashhad University of Medical Sciences, Mashhad, Iran; ^2^Department of Cardiovascular Surgery, Faculty of Medicine, Mashhad University of Medical Sciences, Mashhad, Iran; ^3^Clinical Research Development Unit, Imam Reza Hospital, Faculty of Medicine, Mashhad University of Medical Sciences, Mashhad, Iran; ^4^Department of Anesthesiology, Mashhad University of Medical Sciences, Mashhad, Iran

**Keywords:** Diabetes Mellitus Type II, Glargine Insulin, Coronary Artery Bypass Grafting

## Abstract

***Introduction:*** In this trial, effects of glargine on hyperglycemia in patients with diabetes mellitus type II who were undergoing off-pump coronary artery bypass graft (CAGB), were examined.

***Methods:*** Seventy diabetic patients who were candidate for off-pump CABG were randomly divided into the following two groups (1) Control group who were treated with normal saline+regular insulin and (2) Glargine group who received glargine+regular insulin. Normal saline and glargine were administered subcutaneously 2 hours before surgery, and regular insulin was injected before, during and after the surgery in the intensive care unit (ICU) in both groups. Finally, levels of blood sugar before, 2 hours after starting the surgery and at the end of the surgery, were recorded. Blood sugar levels during ICU stay were also measured every 4 hours for 36 hours.

***Results:*** There were no significant differences in blood sugar levels between the groups at the three time points (i.e. before, 2 hours after starting the surgery and at the end of the surgery). In addition, during 36 hours of ICU stay, blood sugar levels did not show significant variations between the groups; however, 20 hours after ICU admission, blood sugar level was significantly higher in the glargine group (*P*=0.04).

***Conclusion:*** The results indicated that both glargine and regular insulin effectively control the blood glucose in diabetic patients undergoing CABG. However, the blood sugar fluctuation was less in the glargine group than control group.

## Introduction

 Diabetes mellitus is a global epidemic and one of four major non-communicable diseases with increasing prevalence. The World Health Organization estimates that by 2025, 5.4% of the world population will have diabetes.^1^ Diabetes increases the risks of coronary artery disease. Studies estimated a prevalence of 30-40% for diabetes mellitus in patients undergoing coronary artery bypass grafting (CABG).^2,3^ Diabetic patients undergoing CABG have a higher risk of postoperative morbidity and mortality, stroke, renal failure, infections, recurrent episodes of angina and readmission for cardiac-related issues or other causes. So that, increases in blood glucose levels before, during and immediately after surgery predict elevated perioperative complications in patients undergoing cardiovascular surgery; therefore, hyperglycemia is regarded as an indicator of poor outcomes among these patients.^4-7^ Based on the available evidence, reducing perioperative complications and improvement of short- and long-term survival can be achieved by glycemic control in diabetic patients undergoing CABG.^4,6,8^

 Insulin is a peptide hormone that regulates the level of glucose in the blood and it is produced by beta cells of the pancreatic islets. Administeration of exogenous insulin is essential to manage type 1 and, in some cases, type 2 diabetes.^9,10^ There are various forms of insulin that differ in onset and half-life of action; these include short- (e.g. regular), rapid- (e.g. aspart, lispro, and glulisine), intermediate- (e.g. Neutral Protamine Hagedorn (NPH)) and long-acting insulin (e.g. ultralente, glargine, and detemir) as well as mixed insulins.^4,11^

 Since regular and NPH insulins were not a suitable alternative to basal insulin, insulin analogues were introduced.^12,13^ Glargine is a long-acting recombinant insulin.^14^ It binds to the insulin receptor, has an onset of action of 2 hours and a duration of action of 24 hours, with no peak action period.^15,16^ Following subcutaneous injection of glargine as basal insulin once daily, blood glucose level is reduced without causing hypoglycemia.^2,17^ It was shown that in patients undergoing CABG surgery, glargine in combination with continuous insulin infusion better controls blood glucose with no increased side effect.^18,19^

 However, there are few studies on the use of glargine in diabetics undergoing CABG surgery. Therefore, in the present double-blind, randomized clinical trial, effects of glargine and regular insulin on hyperglycemia were compared with regular insulin alone in diabetics undergoing off-pump CABG.

## Materials and Methods

###  Study design

 This randomized, controlled double-blind clinical trial was approved by the Ethics Committee of the Mashhad University of Medical Sciences, Mashhad, Iran (IR.MUMS.MEDICAL.REC.1398.595), and registered at Iranian Registry of Clinical Trial Trials (IRCT20130428013159N11).

 Eighty patients with diabetes mellitus type II who were candidates for off-pump CABG and referred to Ghaem Hospital affiliated to Mashhad University of Medical Sciences, Mashhad, Iran, between June 2020 and November 2021, were screened for eligibility to be enrolled in the present study. Eventually, 70 patients were selected based on the inclusion criteria and completed the study.

 The inclusion criteria were (1) having diabetes mellitus type II, (2) being 35–75 years old, (3) being candidate for off-pump CABG, and (4) being within ASA physical status classification II. The exclusion criteria were (1) having blood sugar > 300 mg/dl or < 150 mg/dl in the morning of the surgery, (2) having a history of previous cardiac operation, on-pump CABG, valve involvement, fever, trauma, carotid intervention, liver and renal diseases, lung disease or digestive disease, or (3) having allergic reaction to glargine, (4) having diabetes mellitus type I.

**Figure 1:**
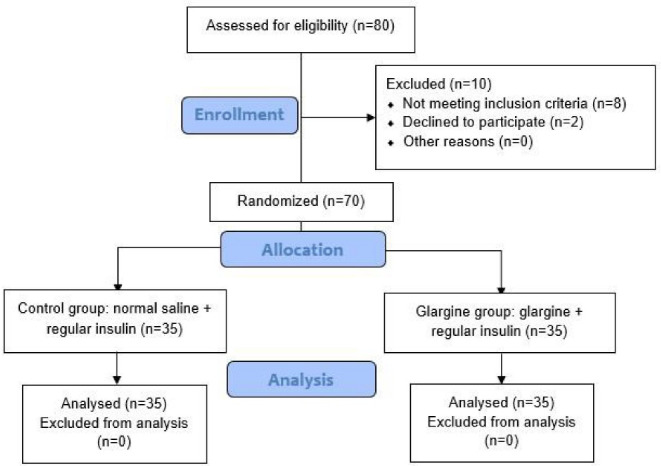
CONSORT flow diagram of the trial

 The patients were undergoing treatment with antidiabetic drugs and they had controlled diabetes. In addition, all operations were elective and non-emergency. In this double-blind study, patients and data collectors were blinded to the treatments/grouping. Written informed consent was obtained from each patient prior to enrollment. Flowchart of the patients selection (CONSORT flow diagram) is illustrated in Figure 1.

###  Randomization and interventions

 Seventy eligible patients were randomized in the following two groups using simple randomization:

1. Control group (n = 35) which received normal saline 0.9% (0.1 ml, subcutaneously, 2 hours before the surgery) + intravenous injection of regular insulin (LANSULIN R VIAL 100 IU/ml, Exir Pharma Co, Iran) before and during the surgery and the postoperative period (according to a modified Van den Berghe protocol accepted in cardiac surgery department of Ghaem hospital).
2. Glargine group (n = 35) which received a single dose of insulin glargine (LANTUS SoloStar® pen, 100 Units/mL, Sanofi-aventis Pharma Co, Germany, (0.5 unit/kg, subcutaneously in the thigh area, 2 hours before the surgery at 37 C °)) + intravenous injection of regular insulin before and during surgery and during the postoperative period (according to a modified Van den Berghe protocol accepted in cardiac surgery department of Ghaem hospital).

In both groups, regular insulin was intravenously injected for blood sugar control, according to a modified Van den Berghe protocol accepted in cardiac surgery department of Ghaem hospital. Based on this protocol,

A) Before surgery; 

 If blood sugar level of patient was ≥ 150 mg/dl then, 2 units of regular insulin was injected for every 50 units increase in blood sugar.

B) During surgery; 

 The unit of regular insulin injection was calculated as follows;

 (Blood sugar of the patient/150)

C) After surgery in intensive care unit (ICU); 

 The unit of regular insulin injection was calculated as follows;

 (Blood sugar of the patient minus 140); which for every 40 units, 4 U regular insulin was injected.

###  Measurement of variables 

 In this trial, several variables including age, sex, body mass index, blood pressure, glomerular filtration rate (GFR), percentage of ejection fraction, hemoglobin A1c (HbA1c), hypoglycemic agents, and duration of surgery were recorded.

 Levels of blood sugar were measured before, 2 hours after starting the surgery and at the end of surgery. In postoperative period, blood sugar changes during the ICU stay were evaluated every 4 hours for 36 hours. Up to 36 hours of monitoring, all patients remained in the ICU in the two groups. Length of hospital stay and mortality during hospitalization were also recorded. Blood sugar goal was within 120–180 mg/dl during the surgery and 120–250 mg/dl after the surgery.^20^

###  Statistical analysis

 Samples sizes calculated using a commercial software package (PASS) with the intent of comparing means μ1 and μ2 from 2 independent groups with variances σ1 and σ2. Based on the findings of Gandhi et al^2^, a sample size of 35 in each group (with α = 0.05, and power = 0.80) was found sufficient.

 Data analysis was done by SPSS version 26.0. The Kolmogorov Smirnov test was applied to evaluate whether the variables were normally distributed. Data are presented as means ( ± standard deviation) or frequencies (percentages). Comparison between glargine and control groups was made using Mann-Whitney test and independent sample T-test (in a normal distribution) for quantitative variables. Categorical variables were analyzed using Chi squared test. Friedman test was used for comparison of blood sugar levels within each group during the ICU stay. A *P* < 0.05 was considered significant for all analyses.

## Results

###  Patient characteristics

 Demographic data in both the groups are shown in Table 1. The average age, graft number, ejection fraction percentage, renal function, duration of surgery, history of hypertension, HbA1c and hypoglycemic agents did not show statistical differences between the two groups. The main criterion for inclusion of the patients in the study was the diabetes mellitus type II and candidate for off-pump CABG, which were similar between groups and patients with diabetes type I were excluded from study. The other variables were controlled at the baseline by randomization method. In addition, performing the surgery by one cardiac surgeon and prescribing drugs in a similar manner and based on the patient’s weight in the both groups led to reduce the effect of confounders.

**Table 1:** Demographic characteristic of the patients

**Variables**			**Glargine group(n=35)**	**Control (n=35)**	***P*** ** value**
Age			66.8 ± 9.5	61.2 ± 11	0.29*
Gender		Male	13(35%)	23(64%)	0.03***
		Female	22(65%)	12(36%)	
Body mass index			26.1 ± 2.3	27.1 ± 4	0.29*
Graft number			2.3 ± 0.4	2.3 ± 0.5	0.7**
Ejection fraction percentage			36.4 ± 10.9	41.4 ± 9.5	0.4*
Glomerular filtration rate			98.2 ± 13.6	98.8 ± 21.5	0.4**
Hemoglobin A1c			6.69 ± 1.10	6.31 ± 1.02	0.147**
Hypertension	Yes		17(50%)	17(50%)	0.99***
	No		18(50%)	18(50%)	
Hypoglycemic agents	Oral agents (Metformin, Glibenclamide, Gliclazide)		23 (65.7%)	25 (71.4%)	0.098***
	Insulin		2 (5.7%)	6 (17.1%)	
	Insulin + oral agents		10 (28.6%)	4 (11.4%)	
Duration of surgery (hours)			3.4 ± 0.6	3.1 ± 0.6	0.07**

Notes: Values are represented as mean ± SD or N (%). ***Pearson Chi Square ** Mann-Whitney Test * Independent Sample T-Test *P* < 0.05 statistically significant.

###  Assessment of blood sugar before and after starting the surgery

 There were no significant differences in blood sugar levels before, 2 hours after starting the surgery and at the end of the surgery between the groups. No significant difference was also observed between the glargine and control groups in mean difference of blood sugar values before and 2 hours after starting the surgery (Table 2).

**Table 2:** Blood sugar levels before and after starting the surgery in the two groups

**Variables**	**Glargine group (n=35)**	**Control group (n=35)**	***P *** **value**
Blood sugar values before the surgery	216 ± 36	232 ± 35	0.16*
Blood sugar values 2 hours after starting the surgery	201 ± 71	187 ± 42	0.9**
Blood sugar values at the end of the surgery	190.7 ± 68.5	198.1 ± 42.7	0.1**
Difference of blood sugar values between Pre- and 2 hours after starting the surgery	18.8 ± 64	44 ± 37	0.6**

Notes: Values are represented as mean ± SD ** Mann-Whitney Test * Independent Sample T-Test *P* < 0.05 statistically significant.

###  Assessment of blood sugar changes during the ICU stay 

 Blood sugar changes during the ICU stay in the two groups are shown in Table 3. There were no significant differences in blood sugar levels during 36 hours of ICU stay between the groups. Only at 20 hours after ICU admission, blood sugar level was significantly higher in the glargine group compared to the control group (*P* = 0.04).

**Table 3:** Blood sugar levels in the two groups during ICU stay

**Variables**	**Blood sugar level (mg/dl)**		***P*** value*
	**Glargine group**	**Control group**	
0 hour	190.7 ± 68.5	198.1 ± 42.7	0.1
4 hour	208.7 ± 77.4	199.5 ± 61.4	0.7
8 hour	235.2 ± 94.3	190.9 ± 53.1	0.09
12 hour	222.5 ± 83.1	189.6 ± 43.8	0.16
16 hour	208.9 ± 70.8	183.5 ± 46.6	0.14
20 hour	207.0 ± 68.7	179.3 ± 30.2	0.04
24 hour	199.3 ± 66.8	194.6 ± 66.3	0.5
28 hour	190.4 ± 59.5	183.5 ± 43.4	0.9
32 hour	172.8 ± 55.0	168.0 ± 28.4	0.6
36 hour	162.1 ± 48.3	181.8 ± 53.1	0.8

Notes: Values are represented as mean ± SD; *Mann-Whitney Test *P* < 0.05 statistically significant.

 In glargine group, the highest and lowest levels of blood sugar were observed 8 and 36 hours after ICU admission, respectively. Besides, the highest and lowest levels of blood sugar in control group, were observed 4 and 32 hours after ICU admission, respectively (Table 3).

 The mean of injected regular insulin during 36 hours of ICU stay in glargine and control groups was 44.2 ± 32 and 39.5 ± 21.4, respectively, which showed no significant difference between the two groups (*P* = 0.8).

 Based on postoperative data, the mean length of hospital stay was 5.17 ± 0.38 days in glargine group and 5.29 ± 0.75 days in control group and there was no significant different between two groups (*P* = 0.993). In addition, no deaths were recorded during hospitalization in both studied groups.

## Discussion

 Considering the importance of diabetes and its known complications in patients undergoing CABG, and due to genetic differences of individuals in drug response in Iranian patients compared to other populations around the world, this study was compared effects of glargine and regular insulin on hyperglycemia with regular insulin alone in patients with diabetes mellitus type II undergoing off-pump CABG. Our study showed blood sugar control of patients with both insulin glargine and regular insulin and reducing the patient’s blood sugar fluctuation in the glargine group compared to control group.

 The main characteristic of type I diabetes is the destruction of insulin-producing pancreatic β-cells so that these patients cannot make insulin. On the other, insulin glargine is a long-acting insulin and it acts longer than regular insulin. Therefore, present study was conducted only on patients with type II diabetes due to reduce the risk of possible hypoglycemia and protection of patients.

 High prevalence of diabetes in patients that require CABG results in increased sternal wound infections, hospital stay length, and postoperative morbidity and mortality.^21,22^ Many studies have indicated that hyperglycemia management in these patients reduces morbidity and mortality and improves short- and long-term survival.^23,24^

 There is no specific guideline for glycemic control in patients with and without diabetes undergoing cardiac surgical procedures.^23^ However, some investigations have shown that control of blood glucose can be achieved by continuous insulin infusion in diabetic patients undergoing CABG.^24,25^

 Insulin glargine is a long-acting insulin subcutaneously used once a day to create a basal level of insulin. It may be combined with rapid-acting insulin or used alone.^26^ After subcutaneous injection, at physiological pH 7.4, the insulin is neutralized and higher order aggregates of insulin hexamers are formed. Dissociation of the hexamers into insulin monomers is a slow process that produces small amounts of insulin glargine that constantly are released. Because of this gradual process, glargine possesses an almost peakless profile.^2,27^

 In a study, hyperglycemia control (for blood glucose level 80 - 140 mg/dl) using once-daily glargine insulin versus twice-daily NPH/regular insulin in patients after cardiovascular surgery was evaluated. Findings showed that glargine insulin resulted in a good glycemic control with lower incidence of hypoglycemia.^28^ However, there is little evidence about the effect of glargine insulin along with continuous regular insulin injection on blood glucose control in diabetic patients undergoing CABG.

 According to the previous studies, the target blood glucose range recommended by the Society of Thoracic Surgeons and the AACE/ADA consensus was reported between 140 and 180 mg/dL after surgery.^29^ However, no evidence was currently found for the necessity of maintaining blood sugar within goal range of 141–180 mg/dL in critically ill diabetic patients. Studies suggest that it is better to individualizing the treatment target ranges,^30^ because every individual is unique and so genetic differences of individuals in drug response can affect duration of the drug’s effect.

 In the present clinical trial, we aimed to control blood glucose within 120–180 mg/dl during the surgery and 120–250 mg/dl after the surgery. The results of present study showed that both insulin glargine and regular insulin treatment effectively control blood glucose. However, the blood sugar fluctuation was less in the glargine group than control group. Hypoglycemic event was not also observed in the dose of glargine used in the present study. However, it may be occurred by increasing the dose of glargine.

 In study conducted by Forouzannia et al effects of continuous insulin infusion with or without subcutaneous glargine insulin on glycemic control in diabetic patients undergoing CABG were evaluated. Treatment of patients with continuous insulin infusion along with 15 units of subcutaneous glargine insulin led to a better blood glucose control compared to continuous human insulin infusion only. In addition, hypoglycemia frequency (blood glucose values < 70 mg/dl) was 0.66%. In this trail, the length of stay in the hospital did not differ significantly between groups.^19^

 Regular insulin requirement was not reduced in the dose of glargine used in the present study. However, it may be decreased by increasing the dose of glargine. In a prospective randomized control study, subcutaneously injection of 1 unit/kg glargine 2 h before surgery was decreased the regular insulin requirement in glargine group compared to control group who received normal saline and human insulin infusion.^2^

 Previously, in two prospective studies, it was shown that perioperative continuous intravenous insulin infusion in diabetic patients undergoing cardiac surgery could improve both the incidence of deep sternal wound infection and mortality.^24,31^

 Concerning the effectiveness of insulin therapy on the mortality in adult patients with a critical illness, Pittas et al in a meta-analysis that included 35 trials, reported that postoperative blood glucose control resulted in beneficial effects on short-term mortality.^32^ Besides, some studies showed that diabetic control in patients undergoing cardiac surgery resulted in shorter length of ICU stay, and lower rate of mortality and infection.^33-35^ On the other, De La Rosa et al showed that mortality did not decrease following strict blood glucose control in patients admitted to ICU.^36^ In our study, there was no significant different in length of hospital stay between two groups and no deaths were recorded in this period.

 There were also some limitations in this study. (1) This study focused only on the effects of glargine insulin on blood sugar level in diabetic patients undergoing CABG. Therefore, evaluation of other parameters related to glucose management in these patients such as short-term and long-term complications, infection are recommended in future studies. (2) Also, in this study, the rational sample size supports the confidence level of the outcomes, however, conducting a multicenter study with a larger sample size is crucial before generalizing the results. (3) As it is well known, it is difficult to measure blood sugar at shorter intervals due to increase in costs and time. Therefore, future studies are recommended to assess blood sugar at shorter intervals during surgery.

## Conclusion

 The findings of the present study showed the effectiveness of both insulin glargine and regular insulin treatment to control blood glucose in diabetic patients undergoing CABG. However, the blood sugar fluctuation was less in the glargine group than control group.

## Acknowledgments

 Authors express their appreciation for financial and moral support provided by the Vice Chancellor for Research, Mashhad University of Medical Sciences. The authors would like to appreciate the Clinical Research Development Unit, Imam Reza Hospital, Mashhad University of Medical Sciences, for their assistance in this manuscript.

## Competing Interest

 The authors do not declare any conflict of interest

## Ethical Approval

 This study was approved by the Ethics Committee of Mashhad University of Medical Sciences (IR.MUMS.MEDICAL.REC.1398.595) and the signed informed consent was obtained from all participants.

## Funding

 This study was supported by Research Council of Mashhad University of Medical Sciences, Mashhad, Iran (Code: 980079), and this body took full responsibility for the financing of the project. The results of the present study are part of thesis of Seyedeh Mahsa Kalati.
